# Nilvadipine in mild to moderate Alzheimer disease: A randomised controlled trial

**DOI:** 10.1371/journal.pmed.1002660

**Published:** 2018-09-24

**Authors:** Brian Lawlor, Ricardo Segurado, Sean Kennelly, Marcel G. M. Olde Rikkert, Robert Howard, Florence Pasquier, Anne Börjesson-Hanson, Magda Tsolaki, Ugo Lucca, D. William Molloy, Robert Coen, Matthias W. Riepe, János Kálmán, Rose Anne Kenny, Fiona Cregg, Sarah O'Dwyer, Cathal Walsh, Jessica Adams, Rita Banzi, Laetitia Breuilh, Leslie Daly, Suzanne Hendrix, Paul Aisen, Siobhan Gaynor, Ali Sheikhi, Diana G. Taekema, Frans R. Verhey, Raffaello Nemni, Flavio Nobili, Massimo Franceschi, Giovanni Frisoni, Orazio Zanetti, Anastasia Konsta, Orologas Anastasios, Styliani Nenopoulou, Fani Tsolaki-Tagaraki, Magdolna Pakaski, Olivier Dereeper, Vincent de la Sayette, Olivier Sénéchal, Isabelle Lavenu, Agnès Devendeville, Gauthier Calais, Fiona Crawford, Michael Mullan, for the NILVAD Study Group

**Affiliations:** 1 Mercer's Institute for Research on Ageing, St. James's Hospital, Dublin, Ireland; 2 Department of Medical Gerontology, Trinity College Dublin (TCD), Dublin, Ireland; 3 Centre for Support and Training in Analysis and Research (CSTAR), University College Dublin (UCD), Dublin, Ireland; 4 School of Public Health, Physiotherapy and Sport Science, University College Dublin (UCD), Dublin, Ireland; 5 Department of Age Related Healthcare, Tallaght Hospital, Dublin, Ireland; 6 Department of Medical Gerontology, Trinity College Dublin, Dublin, Ireland; 7 Department of Geriatric Medicine, Radboudumc Alzheimer Center, Donders Institute of Medical Neurosciences, Radboudumc, Nijmegen, the Netherlands; 8 Division of Psychiatry, University College London and King’s College London, London, United Kingdom; 9 CHU Lille, Univ. Lille, DISTALZ Laboratory of Excellence, F-59000 Lille, France; 10 Department of Psychiatry and Neurochemistry, Institute of Neuroscience and Physiology, Sahlgrenska Academy, University of Gothenburg, Gothenburg, Sweden; 11 Papanikolaou General Hospital of Thessaloniki, Thessaloniki, Greece; 12 Laboratory of Geriatric Neuropsychiatry, IRCCS Istituto di Ricerche Farmacologiche Mario Negri, Milano, Italy; 13 University College Cork Centre for Gerontology and Rehabilitation, Cork, Ireland; 14 Department of Geriatrics and Old Age Psychiatry, Psychiatry II, Ulm University at BKH Günzburg, Günzburg, Germany; 15 Department of Psychiatry, University of Szeged, Szeged, Hungary; 16 Health Research Institute, University of Limerick, Limerick, Ireland; 17 Mathematics Applications Consortium for Science and Industry (MACSI), Department of Mathematics and Statistics, University of Limerick, Limerick, Ireland; 18 Department of Old Age Psychiatry, King's College London, London, United Kingdom; 19 IRCCS Istituto di Ricerche Farmacologiche Mario Negri, Milano, Italy; 20 Pentara Corporation, Salt Lake City, Utah, United States of America; 21 Department of Neurology, University of Southern California, Los Angeles, California, United States of America; 22 Molecular Medicine Ireland (MMI), Dublin, Ireland; 23 Department of Geriatric Medicine, Rijnstate Hospital, Arnhem, the Netherlands; 24 Department of Psychiatry and Neuropsychology, School of Mental Health and Neuroscience, Alzheimer Center Limburg, Maastricht University, Maastricht, the Netherlands; 25 IRCCS Don Gnocchi Foundation-University of Milan, Milan, Italy; 26 Department of Neuroscience (DINOGMI), University of Genoa, Genoa, Italy; 27 IRCCS AOU Polyclinic, Hospital San Martino, Genoa, Italy; 28 Neurology Department, Multimedica, Castellanza, Italy; 29 Centro San Giovanni di Dio—IRCCS Fatebenefratelli, Brescia, Italy; 30 Aristotle University of Thessaloniki (AUTH), First Psychiatric Department, Papageorgiou General Hospital, Thessaloniki, Greece; 31 Ahepa University General Hospital of Thessaloniki, Thessaloniki, Greece; 32 Centre Hospitalier de Calais, Calais, France; 33 Centre Hospitalier Universitaire de Caen, Caen, France; 34 Centre Hospitalier de Lens, Lens, France; 35 Centre Hospitalier de Béthune, Béthune, France; 36 Centre Hospitalier Universitaire d'Amiens, Amien, France; 37 Groupement des Hôpitaux de l'Institut Catholique de Lille (GHICL), Lille, France; 38 Archer Pharmaceuticals, Sarasota, Florida, United States of America; 39 Roskamp Institute, Sarasota, Florida, United States of America; University of Cambridge, UNITED KINGDOM

## Abstract

**Background:**

This study reports the findings of the first large-scale Phase III investigator-driven clinical trial to slow the rate of cognitive decline in Alzheimer disease with a dihydropyridine (DHP) calcium channel blocker, nilvadipine. Nilvadipine, licensed to treat hypertension, reduces amyloid production, increases regional cerebral blood flow, and has demonstrated anti-inflammatory and anti-tau activity in preclinical studies, properties that could have disease-modifying effects for Alzheimer disease. We aimed to determine if nilvadipine was effective in slowing cognitive decline in subjects with mild to moderate Alzheimer disease.

**Methods and findings:**

NILVAD was an 18-month, randomised, placebo-controlled, double-blind trial that randomised participants between 15 May 2013 and 13 April 2015. The study was conducted at 23 academic centres in nine European countries. Of 577 participants screened, 511 were eligible and were randomised (258 to placebo, 253 to nilvadipine). Participants took a trial treatment capsule once a day after breakfast for 78 weeks. Participants were aged >50 years, meeting National Institute of Neurological and Communicative Disorders and Stroke/Alzheimer’s disease Criteria (NINCDS-ADRDA) for diagnosis of probable Alzheimer disease, with a Standardised Mini-Mental State Examination (SMMSE) score of ≥12 and <27. Participants were randomly assigned to 8 mg sustained-release nilvadipine or matched placebo. The a priori defined primary outcome was progression on the Alzheimer's Disease Assessment Scale Cognitive Subscale-12 (ADAS-Cog 12) in the modified intention-to-treat (mITT) population (*n* = 498), with the Clinical Dementia Rating Scale sum of boxes (CDR-sb) as a gated co-primary outcome, eligible to be promoted to primary end point conditional on a significant effect on the ADAS-Cog 12. The analysis set had a mean age of 73 years and was 62% female. Baseline demographic and Alzheimer disease–specific characteristics were similar between treatment groups, with reported mean of 1.7 years since diagnosis and mean SMMSE of 20.4. The prespecified primary analyses failed to show any treatment benefit for nilvadipine on the co-primary outcome (*p* = 0.465). Decline from baseline in ADAS-Cog 12 on placebo was 0.79 (95% CI, −0.07–1.64) at 13 weeks, 6.41 (5.33–7.49) at 52 weeks, and 9.63 (8.33–10.93) at 78 weeks and on nilvadipine was 0.88 (0.02–1.74) at 13 weeks, 5.75 (4.66–6.85) at 52 weeks, and 9.41 (8.09–10.73) at 78 weeks. Exploratory analyses of the planned secondary outcomes showed no substantial effects, including on the CDR-sb or the Disability Assessment for Dementia. Nilvadipine appeared to be safe and well tolerated. Mortality was similar between groups (3 on nilvadipine, 4 on placebo); higher counts of adverse events (AEs) on nilvadipine (1,129 versus 1,030), and serious adverse events (SAEs; 146 versus 101), were observed. There were 14 withdrawals because of AEs. Major limitations of this study were that subjects had established dementia and the likelihood that non-Alzheimer subjects were included because of the lack of biomarker confirmation of the presence of brain amyloid.

**Conclusions:**

The results do not suggest benefit of nilvadipine as a treatment in a population spanning mild to moderate Alzheimer disease.

**Trial registration:**

Clinicaltrials.gov NCT02017340, EudraCT number 2012-002764-27.

## Abstract 2

In a randomised controlled trial, Brian Lawlor and colleagues investigate whether the blood pressure medication nivaldipine can slow the progression of Alzheimer disease.

## Author summary

**Why was this study done?:**

- There are few licensed drug treatments for Alzheimer disease and none are effective in slowing the rate of disease progression.
- Nilvadipine is a licensed blood pressure medication and has been shown to lower brain amyloid and improve memory function in animal models of Alzheimer disease.
- If nilvadipine were shown to be effective in slowing the rate of progression of Alzheimer disease, because it is already licensed and available to treat high blood pressure, it would be possible to introduce the drug for use in Alzheimer disease relatively quickly.

**What did the researchers do and find?:**

- We carried out an investigator-led clinical trial funded by the European Union across 23 academic university sites and involving 511 patients with mild- and moderate-stage Alzheimer disease, as diagnosed by a clinician.
- We tested whether a single dose of nilvadipine, compared with placebo, was safe and slowed the progression of Alzheimer disease over a period of 18 months.
- We found that nilvadipine appeared safe and was well tolerated but did not slow decline in cognition or function in this group of mild- and moderate-stage Alzheimer disease patients.

**What do these findings mean?:**

- Nilvadipine does not appear to be effective as a treatment for people with mild- or moderate-stage Alzheimer disease.
- We cannot rule out that this medication may help at an earlier stage of the disease process, before the person experiences loss of function.

## Introduction

Observational studies have suggested a benefit of certain blood pressure medications on reducing the risk of developing dementia [1]. Particular antihypertensive agents have also been shown to decrease Alzheimer disease pathology in the brains of people with hypertension, independently of blood pressure control, suggesting a direct effect of these medications against the biological processes underpinning Alzheimer disease [2,3]. One antihypertensive, for which there is clinical and scientific rationale for disease-modifying efficacy in Alzheimer disease, is nilvadipine. Nilvadipine is a dihydropyridine (DHP) calcium channel blocker and is licensed in a number of countries to treat patients with hypertension. Nilvadipine is reported to have a number of neuroprotective mechanisms of action other than direct calcium channel blockade and maintenance of intracellular calcium homeostasis, including lowering Amyloid beta 40 and 42 amino acid peptides (Aβ40 and Aβ42) production in vitro and in vivo in transgenic mouse models of Alzheimer disease, and enhancing Aβ clearance across the blood–brain barrier in in vivo mouse models [4,5]. However, many other DHPs do not share these properties and some may actually increase Aβ40 and Aβ42 production in vitro [4], demonstrating that amyloid lowering is not a class effect of DHPs. In addition to effects on Aβ production and clearance, nilvadipine specifically has also shown efficacy against a broad range of other putative Alzheimer disease pathological mechanisms, including tau-phosphorylation, reduced cerebral blood flow, and neuroinflammation [6–9].

In clinical studies, nilvadipine stabilised cognitive decline and reduced conversion to Alzheimer disease in a small study of patients with hypertension and mild cognitive impairment [10]. Another 6-week open label study demonstrated that nilvadipine was safe and well tolerated in patients with Alzheimer disease and did not reduce blood pressure in nonhypertensive patients with Alzheimer disease, but appropriately lowered blood pressure in hypertensive cases [11].

These studies are complemented by a number of epidemiological and interventional studies involving different calcium channel blockers that have reported on the potential benefit of this drug class in the prevention of Alzheimer disease. In the treatment of Systolic Hypertension in Europe (Syst-Eur) trial, which involved over 2,400 older participants with systolic hypertension treated with the DHP calcium channel blocker, nitrendipine, there was a reported 55% reduction in the incidence of Alzheimer disease [12,13]. The Baltimore Longitudinal Study of Aging found a nonsignificant apparent benefit towards reduced relative risk of Alzheimer disease in patients treated with DHP calcium channel blockers, with no lowered risk observed in the non-DHP calcium channel blocker treatment group [14].

To our knowledge, there has been no definitive intervention study with a calcium channel blocker to test for an effect on slowing the rate of cognitive decline in patients with Alzheimer disease.

Given the previous preclinical and clinical data suggesting the potential efficacy for nilvadipine and related compounds against Alzheimer disease, the objective of this 78-week randomised, placebo-controlled study was to determine whether treatment with nilvadipine sustained-release 8 mg, once a day, was effective and safe in slowing the rate of cognitive decline in patients with mild to moderate Alzheimer disease.

## Methods

### Study design

This 18-month Phase III, randomised, placebo-controlled, double-blind, parallel-group study was carried out at 23 academic centres in nine European countries: Ireland (two sites), United Kingdom (one site), Italy (four sites), the Netherlands (three sites), France (seven sites), Greece (three sites), Sweden (one site), Germany (one site), and Hungary (one site) (S1 Table). The trial project office was based at St. James’s Hospital, Dublin, Ireland, which was also the sponsor. The trial coordinating institution was Trinity College, University of Dublin, and the trial was funded by the European Commission, under a Framework 7 Programme Health Theme collaborative project grant. The trial database, randomisation, and allocation system were maintained by the Clinical Trials Unit at King’s College London, and the statistical analysis was conducted at the University College Dublin Centre for Support and Training in Analysis and Research (UCD CSTAR). As part of the overall governance of the trial, there was a Scientific Advisory Board, an independent Ethics Advisory Board, and an independent Data Safety Monitoring Board. Approval of the study protocol and all related documents was obtained from the appropriate National Competent Authorities, Independent Ethics Committees, and Institutional Review Boards for all study sites. Additional information is provided below and in supplementary files S1 Text (study design and treatment), S2 Text (detailed statistical methods), and S3 Text (trial-associated boards).

### Participants

A detailed list of inclusion and exclusion criteria is provided in the published protocol [15]. Briefly, participants were aged >50 years, meeting National Institute of Neurological and Communicative Disorders and Stroke/Alzheimer’s disease Criteria (NINCDS-ADRDA) for diagnosis of probable Alzheimer disease [16], with a Standardised Mini-Mental State Examination (SMMSE) [17] score of ≥12 and <27, and having a caregiver available to complete relevant assessment instruments. If on a cholinesterase inhibitor or memantine, the dose had to be stable for >12 weeks. People with dementia because of other causes or with known sensitivity to calcium channel blockers were excluded.

All participants provided written informed consent before enrolling in the study. The consent form was amended as required in each country to comply with local ethics requirements. All caregivers also provided consent for involvement.

### Randomisation and masking

Participants were randomly assigned to nilvadipine sustained-release 8 mg or placebo using block randomisation with randomly varying block sizes, stratified by site, using an online system integrated with stock control across sites. Participants, caregivers, and assessors were blinded to treatment assignment.

### Procedures

Participants took a trial treatment capsule once a day after breakfast for 78 weeks and returned their used treatment boxes at subsequent dispensing visits, when the number of returned capsules was recorded. Participants were assessed at 6, 13, 26, 39, 52, 65, and 78 weeks after commencing treatment. Participants were followed up 4 weeks after the final, week 78 visit.

### Outcomes

The co-primary outcome measures were the change from baseline in the 12-item Alzheimer Disease Assessment Scale–Cognitive Subscale (ADAS-Cog 12) [18] and the Clinical Dementia Rating scale sum of boxes (CDR-sb) [19]. The key secondary outcome measure was the Disability Assessment for Dementia (DAD) [20], as maintenance of functional abilities is considered a crucial benefit of any potential treatment. Data on all primary and secondary outcome measures were collected at baseline and at 13, 52, and 78 weeks. Safety was assessed through the collection of data on adverse events (AEs), blood pressure, and laboratory tests.

### Statistical analysis

The sample size of 250 patients in each group was calculated to allow detection of a 50% reduction in cognitive decline in the nilvadipine group over the 78 weeks of follow-up [15]. This resulted in 90% power to detect a 3.5-point group difference in the decline in ADAS-Cog 12 (SD = 10), and 81% power to also detect a significant effect on the CDR-sb as a gated co-primary end point. The sample size calculation included allowance for 30% loss to follow-up.

The primary and secondary efficacy analyses were conducted in a modified intention-to-treat (mITT) population, including all participants randomised who had both a baseline assessment and at least one later assessment. The safety set included all patients who took at least one dose of the trial treatment.

A secondary per-protocol analysis was carried out using only those patients compliant with medication (defined as taking >80% of doses) and with all assessments on schedule.

The primary and secondary end point analyses consisted of linear mixed-effects models, with country as a random effect and correlated residuals over time. Findings hinged on a *p*-value less than 0.05 for a (Visit × Arm) interaction test using change scores from baseline and adjusting for the baseline score. We adopted a gated approach to control the false positive rate over multiple end points. The ordered outcomes were as follows: change from baseline of ADAS-Cog 12 (analysed in discrete time); followed by the change in CDR-sb; then, in order, ADAS-Cog 12 and CDR-sb were to be tested for a linear improvement over continuous time. The key secondary outcome of DAD was next on the list, followed by the other secondary outcomes. In the case of a nonsignificant result, any further analyses are purely exploratory, with no further tests of a null hypothesis. Full technical details and description of the gated approach and statistical models are given in the S1 Text file.

Responder analyses were conducted on a dichotomised change score from baseline to week 78 using logistic regression, with no imputation for missing values. Preplanned subgroup analyses included examination of a difference in nilvadipine effect size between mild and moderate Alzheimer disease (≥20 versus <20 on baseline SMMSE, respectively), between males and females, and between Apolipoprotein E gene (*APOE*) ε4 allele carriers and noncarriers. The latter analysis was limited to the patient subgroup that participated in the blood biomarker study [21]. Subgroup differences in efficacy were examined by a three-way interaction of the subgroup with visit and treatment arms.

Baseline and safety end points were tested by standard tests for proportions (Pearson chi-squared test) or rates (Poisson count model), with no corrections applied for multiple testing.

An independent Data Safety Monitoring Board, blind to group assignment, reviewed safety data throughout the trial.

This trial adhered to the Declaration of Helsinki and International Conference on Harmonisation Good Clinical Practice (ICH GCP) guidelines and was conducted in compliance with the protocol, data protection regulations, and all other regulatory requirements, as appropriate.

## Results

### Participants

**Fig 1:**
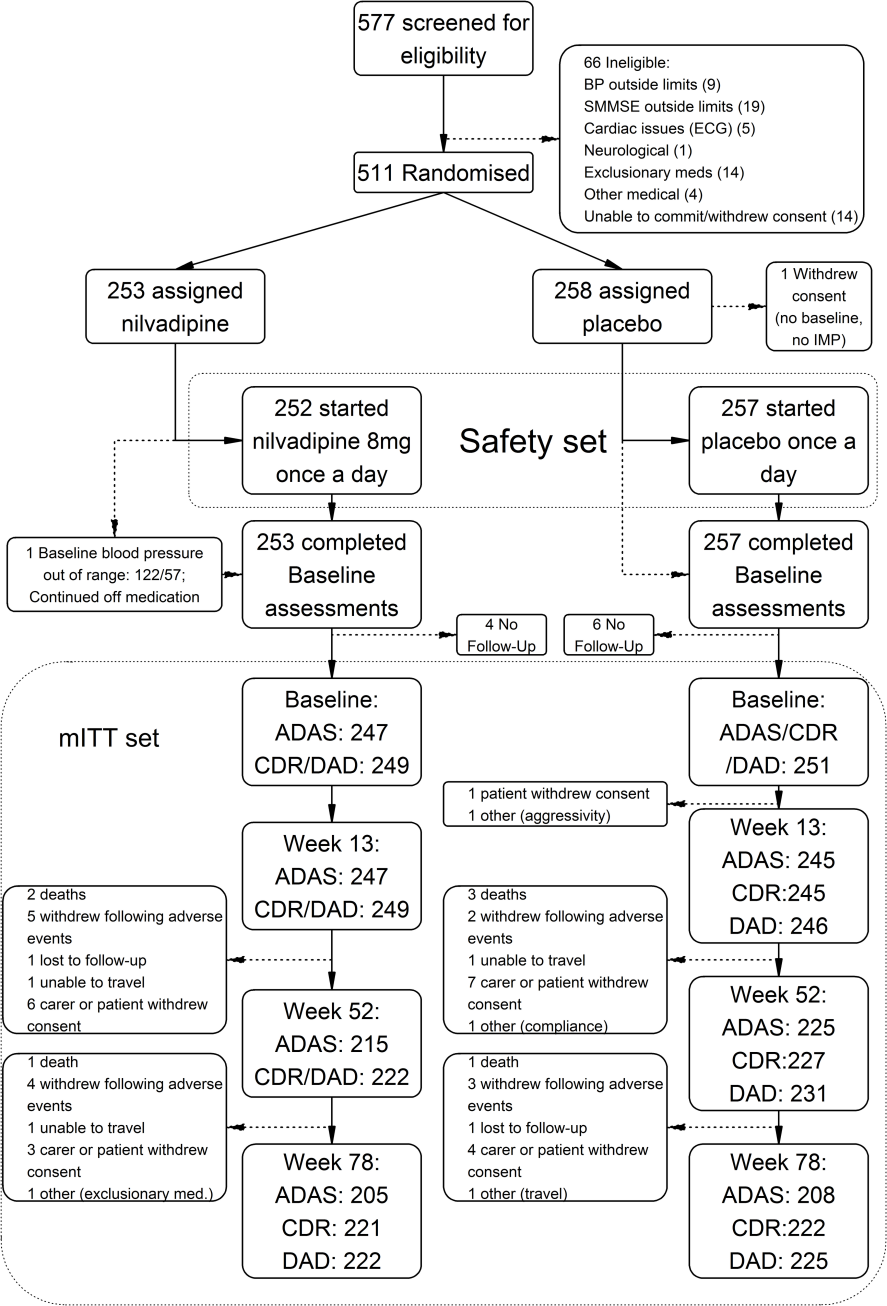
Flowchart of the NILVAD study according to the CONSORT guideline. ADAS, Alzheimer’s Disease Assessment Scale-Cognitive Subscale 12 item; BP, blood pressure; CDR, Clinical Dementia Rating Scale sum of boxes; DAD, Disability Assessment for Dementia; ECG, electrocardiogram; IMP, investigational medicinal product; NILVAD, Nilvadipine in Alzheimer disease; SMMSE, Standardised Mini-Mental State Examination.

Between 15 May 2013 and 13 April 2015, 511 eligible participants were randomised; the last outcomes visit was in November 2016. Of the 511 randomised, 498 had at least one post-baseline ADAS-Cog 12 assessment and comprised the mITT population (Fig 1), with 247 on nilvadipine and 251 on placebo. The proportion of ADAS-Cog 12 assessments completed was high, allowing us to exceed our sample size target (see Fig 1). Trial medication was interrupted by 103 patients during the course of the study (55 nilvadipine, 48 placebo), of whom 4 resumed medication; mean treatment compliance was 88% (capsules taken over days in study), and 80.4% of patients were compliant with assigned medication at a threshold of 80% of capsules taken, balanced between arms.

Baseline demographic and Alzheimer disease–specific characteristics were similar between treatment groups (Table 1, Table 2). There were no significant differences at baseline or end of trial in the prescribing of Alzheimer disease medications (acetylcholinesterase inhibitors and/or memantine) or non-Alzheimer disease concomitant medications (Table 1). Vascular risk factors, notably hypertension, hypercholesterolemia, and kidney disease, were also similar, with the exception of diabetes, which was more common in the nilvadipine group (Table 1). Comorbid medical conditions at baseline were substantially more prevalent in the nilvadipine group than in the placebo group, and predominantly in the endocrine class, which included diabetes (Table 1). *APOE* genotype was available from 161 participants in the nilvadipine group and 167 in the placebo group.

**Table 1:** Characteristics of the modified intention-to-treat sample.

Characteristics		Nilvadipine (*N* = 247)	Placebo (*N* = 251)
Demographics and anthropometrics			
Sex *N* (%)	Female	161 (65%)	147 (59%)
Baseline age (years)	mean (SD)	73.1 (8.66)	72.8 (7.84)
Ethnicity *N* (%)	White	241 (98%)	244 (97%)
	Asian	1 (0.4%)	2 (0.8%)
	Black	1 (0.4%)	4 (1.6%)
	Other	4 (1.6%)	1 (0.4%)
Baseline BMI	mean (SD)	25.3 (3.92)	25.8 (4.45)
Baseline blood pressure	mean (SD) SBP/DBP	138 (14) / 77 (9)	137 (14) / 77 (9)
Week 13 blood pressure	mean (SD) SBP/DBP	131 (15) / 73 (9)	137 (16) / 76 (9)
Week 52 blood pressure	mean (SD) SBP/DBP	131 (15) / 74 (9)	135 (14) / 76 (9)
Week 78 blood pressure	mean (SD) SBP/DBP	132 (16) / 74 (10)	135 (16) / 75 (9)
Baseline vascular risk factors			
Diabetes		28 (12%)	12 (5%)
Kidney disease		3 (1%)	5 (2%)
Hypercholesterolemia		82 (34%)	83 (33%)
Hypertension		82 (34%)	87 (35%)
Baseline blood pressure (median SBP / DBP)		140 / 77	138 / 77
Week 78 blood pressure (median SBP / DBP)		130 / 74	135 / 74
Baseline medical status			
Patients on AD concomitant medications		173 (69%) on 1; 65 (26%) on 2+	170 (66%) on 1; 75 (29%) on 2+
Patients on non-AD concomitant medications		221 (88%)	219 (85%)
Comorbid medical conditions (per patient)	mean (SD)	2.84 (2.09)	2.43 (1.87)

Data are mean (standard deviation), median (IQR: first and third quartiles), *n* (%), or *n*/*N* (%).

Abbreviations: AD, Alzheimer disease; BMI, body mass index; DBP, diastolic blood pressure; IQR, interquartile range; SBP, systolic blood pressure.

**Table 2:** Baseline Alzheimer disease characteristics.

Characteristics	Sample statistics	Nilvadipine (*N* = 247)	Placebo (*N* = 251)
Years since diagnosis	mean (SD)	1.73 (1.66)	1.70 (1.78)
Years since symptom onset	mean (SD)	4.31 (2.56)	4.28 (2.72)
*APOE* ε4 carrier	*n/N* (%)	94/161 (58%)	100/167 (60%)
SMMSE	mean (SD)	20.3 (3.76)	20.5 (3.89)
SMMSE < 20	*N* (%)	93 (38%)	94 (37%)
Baseline ADAS-Cog 12	mean (SD)	34.4 (10.5)	34.5 (10.8)
Baseline CDR-sb (*N* = 249 + 251)	mean (SD)	5.34 (2.76)	5.17 (2.73)
Baseline DAD (*N* = 249 + 251)	mean (SD)	29.7 (8.0)	30.4 (8.1)

Data are mean (standard deviation), *n* (%), or *n*/*N* (%).

Abbreviations: ε4, epsilon 4 allele; ADAS-Cog 12, Alzheimer Disease Assessment Scale–Cognitive Subscale (12 item); *APOE*, Apolipoprotein E gene; CDR-sb, Clinical Dementia Rating sum of boxes; DAD, Disability Assessment for Dementia; SMMSE, Standardised Mini-Mental State Examination.

### Efficacy end points

**Fig 2:**
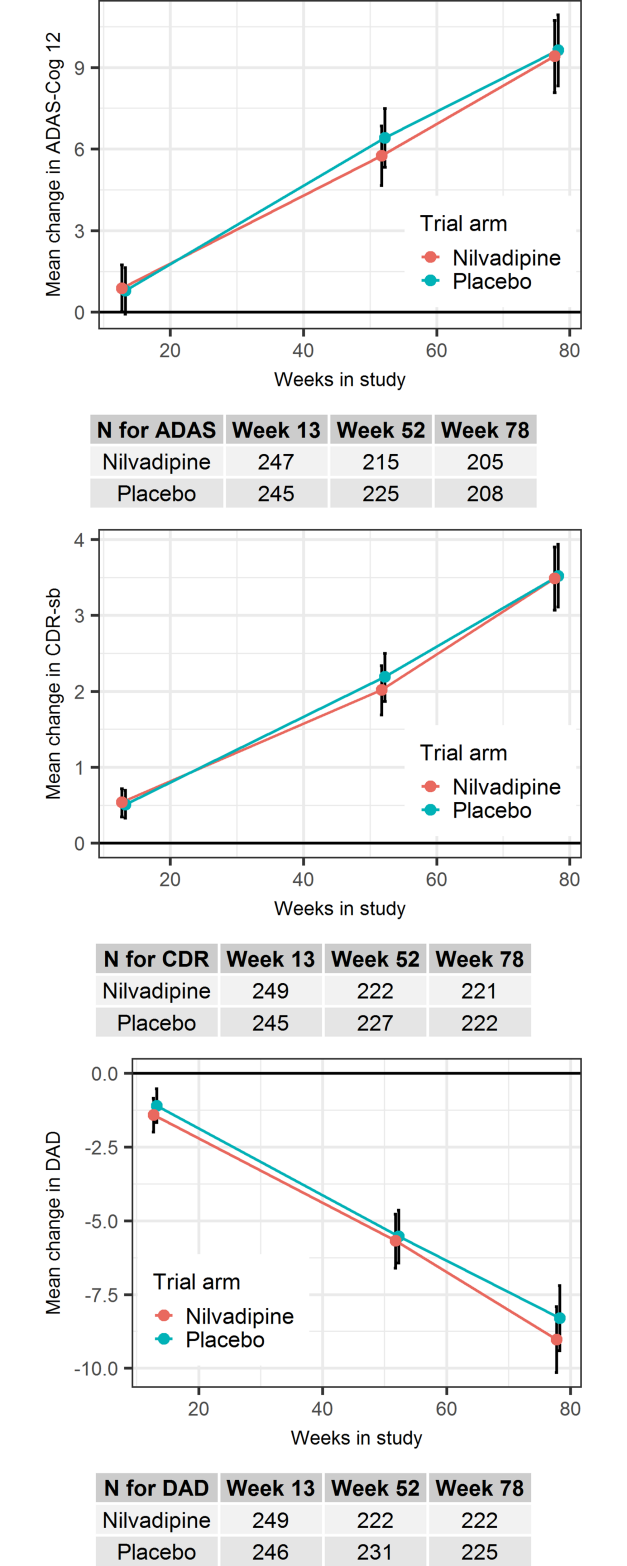
Estimated marginal means and 95% confidence intervals of the per-visit change from baseline in cognition and functional performance, as measured by the primary and key secondary outcomes, respectively. ADAS/ADAS-Cog 12, Alzheimer's Disease Assessment Scale Cognitive-12; CDR/CDR-sb, Clinical Dementia Rating Scale sum of boxes; DAD, Disability Assessment for Dementia.

No treatment effect was observed at a statistically significant level for the first primary outcome analysis (*p* = 0.465). The nilvadipine difference from placebo, in change from baseline in the ADAS-Cog 12 score, was −0.22 (95% CI, −2.01–1.57) (Table 3). Similarly, nilvadipine did not show any clinically meaningful effects on CDR-sb and DAD (Table 3, Fig 2).

**Table 3:** Efficacy analyses for primary outcomes (ADAS-Cog 12 and CDR-sb), and the key secondary outcome (DAD).

Outcomes			Week 0	Week 13	Week 52	Week 78	*p*-value*
ADAS-Cog 12^Ɨ^	Nilvadipine	Mean ± SD	34.4 ± 10.5	35.2 ± 11.3	39.4 ± 13.1	41.9 ± 14.6	
		Δ (95% CI)		0.88 (0.02–1.74)	5.75 (4.66–6.85)	9.41 (8.09–10.73)	
	Placebo	Mean ± SD	34.5 ± 10.8	35.3 ± 11.6	40.3 ± 13.9	41.9 ± 14.5	
		Δ (95% CI)		0.79 (−0.07–1.64)	6.41 (5.33–7.49)	9.63 (8.33–10.93)	
	Group difference			0.09 (−0.96–1.15)	−0.65 (−2.10–0.80)	−0.22 (−2.01–1.57)	0.465
CDR-sb^ǂ^	Nilvadipine	Mean ± SD	5.34 ± 2.76	5.87 ± 2.96	7.29 ± 3.69	8.72 ± 4.60	
		Δ (95% CI)		0.54 (0.35–0.72)	2.02 (1.69–2.34)	3.49 (3.07–3.90)	
	Placebo	Mean ± SD	5.17 ± 2.73	5.71 ± 3.17	7.22 ± 4.02	8.38 ± 4.45	
		Δ (95% CI)		0.51 (0.33–0.70)	2.19 (1.87–2.50)	3.52 (3.11–3.94)	
	Group difference			0.02 (−0.25–0.29)	−0.17 (−0.62–0.28)	−0.04 (−0.62–0.55)	N/A
DAD^§^	Nilvadipine	Mean ± SD	29.7 ± 8.0	28.4 ± 8.2	24.3 ± 10.2	21.1 ± 11.5	
		Δ (95% CI)		−1.42 (−1.99–−0.85)	−5.68 (−6.60–−4.77)	−9.02 (−10.14–−7.91)	
	Placebo	Mean ± SD	30.4 ± 8.1	29.2 ± 8.6	25.1 ± 10.4	22.8 ± 11.3	
		Δ (95% CI)		−1.10 (−1.67–−0.52)	−5.53 (−6.43–−4.64)	−8.30 (−9.40–−7.20)	
	Group difference			−0.32 (−1.13–0.49)	−0.15 (−1.43–1.13)	−0.73 (−2.29–0.84)	N/A¶
ADAS-Cog 12	Placebo time trend (per week)		0.120 (0.109–0.132)				N/A¶
	Nilvadipine change in trend		−0.002 (−0.019–0.014)				
CDR-sb	Placebo time trend (per week)		0.043 (0.040–0.047)				N/A¶
	Nilvadipine change in trend		0.0005 (−0.005–0.005)				

Δ Change from baseline.

*From F test for Visit × Arm interaction term. All models were controlled for baseline measurement and included country as a random effect and unstructured correlation between time points.

^Ɨ^Scores on the ADAS-Cog 12 range from 0 to 80, with higher scores indicating greater cognitive impairment [18].

^ǂ^Scores on the CDR-sb range from 0 to 18, with higher scores indicating worse functioning [19].

^§^Scores on the DAD range from 0 to 100, with higher scores indicating less impairment [20].

¶ After the nonsignificant outcome on the primary outcome ADAS-Cog 12, all other primary and secondary gated outcomes are not judged for significance, as defined by the preplanned analysis.

Abbreviations: ADAS-Cog 12, Alzheimer's Disease Assessment Scale Cognitive-12; CDR-sb, Clinical Dementia Rating Scale sum of boxes; DAD, Disability Assessment for Dementia.

Per-protocol analyses showed identical patterns to the primary analysis. The prespecified responder analysis showed no effects of nilvadipine on the proportion of patients maintaining cognition or function as measured by the ADAS-Cog 12: odds ratio 1.09 (95% CI, 0.65–1.84), the CDR-sb: odds ratio 1.74 (95% CI, 0.99–3.06), or the DAD: odds ratio 0.90 (95% CI, 0.54–1.51).

The predefined subgroup analyses were inspected to identify group differences (S2 Table, S3 Table, S4 Table); we note that no hypothesis tests were performed for these exploratory analyses. Comparing those with mild to those with moderate Alzheimer disease, there was less decline in the mild group on nilvadipine compared to placebo. However, a greater decline was seen in the moderate group treated with nilvadipine. For gender, males showed less decline than females on nilvadipine compared to placebo. Furthermore, *APOE* ε4 allele carriers showed less decline than noncarriers on nilvadipine (S2 Table, S3 Table, S4 Table).

### Safety

Participants who received at least one dose of the study drug comprised the safety population (*n* = 509). Despite a higher total number of AEs or serious adverse events (SAEs) in the nilvadipine group (Table 4) the number of patients with at least one AE or SAE were substantially similar. The median change in systolic blood pressure from baseline to week 78 was −5 mmHg and the number of falls, complaints of dizziness, or syncope were very similar between groups (Table 4). The number of deaths was 10 (7participants died during the study duration and a further 3 during the longer-term follow-up of SAEs). No deaths were judged by the investigators to be related to treatment. Emergent clinically significant blood test results on nilvadipine and placebo from baseline to week 78 were too rare to draw conclusions but were not elevated in the nilvadipine group. Between-group differences were observed on aggregated significant and nonclinically significant abnormal blood markers; these reflected more elevated results on placebo at trial end for creatinine (9%–13%) and calcium (7%–11%), or fewer elevated results on nilvadipine for mean corpuscular volume (MCV) results (10%–7%) (S5 Table). A comparison of the Medical Dictionary for Regulatory Activities (MedDRA)-coded AEs (S6 Table) showed small differences (<6%) between groups for the following events: fall (worse on placebo), cough, cellulitis, peripheral edema, insomnia, and hypotension.

**Table 4:** Safety end points.

Characteristics	Nilvadipine (*N* = 252)	Placebo (*N* = 257)
****Adverse Events****		
Total logged	1,129	1,030
Possibly, Probably, or Definitely related to IMP	223	178
Patients	206 (82%)	201 (78%)
Patients with Possibly, Probably, or Definitely related	142 (56%)	145 (56%)
Patients with Dizziness	30 (12%)	29 (11%)
Patients with Fall	40 (16%)	38 (15%)
Patients with Fracture	16 (6%)	9 (4%)
Patients with Peripheral edema	15 (6%)	3 (1%)
Patients with Syncope	12 (5%)	10 (4%)
****Serious Adverse Events****		
Total logged	146	101
Possibly, Probably, or Definitely related to IMP	17	19
Patients	50 (20%)	42 (16%)
Patients with Possibly, Probably, or Definitely related	7 (3%)	9 (4%)
**Mortality**^Ɨ^	3 (1.2%)	4 (1.6%)

^Ɨ^Three further individuals died at or after week 82 of the study (all in the placebo group).

Note that patients are counted if they had one or more events of the type listed.

Abbreviations: CDR, CDR-sb (Clinical Dementia Rating sum of boxes); DAD, Disability Assessment for Dementia; DBP, diastolic blood pressure; IMP, investigational medicinal product; mITT, modified intention-to-treat; SBP, systolic blood pressure; SMMSE, Standardised Mini-Mental State Examination.

## Discussion

To our knowledge, this is the first definitive intervention study of nilvadipine, a DHP calcium channel blocker with demonstrated Aβ-lowering properties in animal studies, for the treatment of Alzheimer disease. The results of this study indicated no benefit of nilvadipine as a treatment in a population spanning mild to moderate Alzheimer disease. There were no obvious methodological limitations that could have contributed to these negative findings for the primary and secondary outcomes in the overall treatment population. Recruitment was to target, the dropout and missing data rates were low. The rate of decline in the placebo group on the ADAS-Cog 12 was consistent with previous Phase III clinical trials involving mild to moderate Alzheimer disease participants. Treatment and placebo arms were well balanced, although there were more patients with abnormal glucose levels and with diabetes in the nilvadipine group at baseline. The higher frequency of diabetes in the nilvadipine group is unlikely to have had a bearing on the overall negative finding, as the effect of diabetes on cognitive decline in established Alzheimer disease is unclear [22]. Furthermore, data from a sub-study confirm that there was no significant imbalance between the nilvadipine and the placebo groups in terms of antihypertensive use (J. Claassen & M.G.M. Olde Rikkert, personal communication, see S4 Text).

The overall safety and AE profile for nilvadipine was favourable in this older population. There was no significant difference in the number of deaths, AEs, or SAEs that could be attributed to treatment. Blood pressure effects were modest, with only a median 5 mmHg drop in systolic blood pressure from baseline to week 78 in the nilvadipine treated group.

The findings from the predefined subgroup analyses suggest differential effects of nilvadipine in those at a milder disease stage, in *APOE* ε4 allele carriers, and in males. However, no significance tests were conducted on these subgroups, and these findings will require further investigation to determine if there are specific subgroups within the overall population that respond either positively or negatively to nilvadipine treatment. For instance, consistent with other anti-amyloid treatment trials suggesting that milder patients may respond better [23], in these exploratory analyses, those with an SMMSE >20 appeared to decline at a slower rate than those with an SMMSE <20. However, greater decline on the ADAS-Cog 12 in moderate-stage patients on nilvadipine treatment should also be noted. Similarly, the gender and *APOE* ε4 allele carrier results warrant further exploration, although the number of patients participating in the *APOE* study (64%) was fewer than the overall treatment population. Further exploratory analyses, making use of the sub-study data, will look for correlation between biomarkers (in both blood and cerebrospinal fluid [CSF]), cerebral blood flow, and other brain imaging data to better understand whether specific mechanisms, e.g., via a blood pressure–lowering pathway or changes in Aβ or tau correlate with cognitive change.

The strengths of this investigator-driven clinical trial include the successful recruitment and retention of participants and the conduct of the study to a high standard. There are, however, a number of issues related to the study design that could be considered for future trials of this nature that are suggested by our main findings. Firstly, a single-dose strategy was used, and it is possible that an insufficient dose was given to effect a treatment response. The side effect profile for nilvadipine in this older, mild to moderate Alzheimer disease population was favourable and the effect on blood pressure quite modest, so it would probably have been safe to give a higher dose. While we predicted that any effect of nilvadipine on cognition would be via an anti-amyloid rather than a blood pressure–lowering pathway, it is possible that a lack of benefit in the overall population may have been contributed to by the modest blood pressure–lowering effect of nilvadipine in this study. Secondly, the lack of biomarker confirmation of the diagnosis of Alzheimer disease, which could mean that up to 20% of patients included in the trial may not have had significant amyloid pathology [24], could be taken into account in the design of future trials of this nature. A third issue to consider is the timing of the intervention in the course of Alzheimer disease. Many anti-amyloid treatments have failed in populations with established mild to moderate Alzheimer disease, and it is a commonly held belief that it may be too late to treat established dementia with amyloid-lowering drugs when there is already associated significant neuronal damage [25]. Similarly, if cerebral hypoperfusion triggers or accelerates the deposition of amyloid pathology, intervention with a drug that can improve cerebral blood flow should occur at the earliest possible stage if it is to be effective as a disease-modifying agent. The latter two limitations reflect the rapidly evolving evidence over recent years since this study was designed, highlighting the ability and necessity of more detailed phenotyping and a focus on earlier-stage intervention. Treatment at the prodromal stage of the Alzheimer disease process might therefore be a more successful point at which to intervene with nilvadipine.

## Conclusions

This study of Nilvadipine at a dose of 8 mg found no overall effect on slowing the rate of cognitive decline in a population spanning mild to moderate Alzheimer disease.

## Supporting information

S1 Text Detailed statistical methods. (DOCX)

S2 Text Study design and treatment. (DOCX)

S3 Text Trial-associated boards. (DOCX)

S4 Text Personal correspondence. (DOCX)

S1 Table Distribution of treatment arms recruited in each site. (DOCX)

S2 Table ADAS-Cog 12 sex subgroup analysis results. ADAS-Cog 12, Alzheimer's Disease Assessment Scale Cognitive-12.
(DOCX)

S3 Table ADAS-Cog 12 *APOE* ε4 subgroup analysis results. ε4, epsilon 4 allele; ADAS-Cog 12, Alzheimer's Disease Assessment Scale Cognitive-12; *APOE*, Apolipoprotein E gene.
(DOCX)

S4 Table ADAS-Cog 12 severity subgroup analysis results. ADAS-Cog 12, Alzheimer's Disease Assessment Scale Cognitive-12.
(DOCX)

S5 Table Clinical chemistry and haematology findings at screening visit and week 78. (DOCX)

S6 Table MedDRA coded adverse events per group. MedDRA, Medical Dictionary for Regulatory Activities.
(XLSX)

S1 CONSORT Checklist (DOCX)

## Abbreviations

Aβ40: Amyloid beta 40 amino acid peptide
Aβ42: Amyloid beta 42 amino acid peptide
ADAS-Cog 12: Alzheimer's Disease Assessment Scale Cognitive Subscale-12
AE: adverse event
APOE: Apolipoprotein E gene
CDR-sb: Clinical Dementia Rating Scale sum of boxes
CSF: cerebrospinal fluid
DAD: Disability Assessment for Dementia
DBP: diastolic blood pressure
DHP: dihydropyridine
ECG: electrocardiogram
ICH GCP: International Conference on Harmonisation Good Clinical Practice
IMP: investigational medicinal product
IQR: interquartile range
MCV: mean corpuscular volume
MedDRA: Medical Dictionary for Regulatory Activities
mITT: modified intention-to-treat
NINCDS-ADRDA: National Institute of Neurological and Communicative Disorders and Stroke/Alzheimer’s disease Criteria
SAE: serious adverse event; SBP, systolic blood pressure
SMMSE: Standardised Mini-Mental State Examination
Syst-Eur: Systolic Hypertension in Europe
UCD CSTAR: University College Dublin Centre for Support and Training in Analysis and Research
