# Supplementary material for: Nilvadipine in mild to moderate Alzheimer disease: A randomised controlled trial
Source: PLoS Med. 2018 Sep 24;15(9):e1002660. doi: 10.1371/journal.pmed.1002660 (PMC6152871; doi:10.1371/journal.pmed.1002660)
Supplement: S1 Text — (DOCX) [file pmed.1002660.s001.docx]

## Study design and treatment

Investigational medicinal product was manufactured in three batches to ensure adequate drug life for the duration of the study. Nilvadipine and placebo were identically overencapsulated by LC2 Pharma. Once a batch of investigational medicinal product was produced with identical treatment packs, with the same batch number and expiry for active and placebo, a treatment pack schedule was sent from the Clinical Trials Unit indicating a unique identification number to be applied to each active and placebo medication pack. A copy of the blinding list was stored within the Clinical Trials Unit in an area on the server accessible only to the Clinical Trial Unit programmer and manager. All study staff remained blind to the treatment allocation until the end of the study. The randomization file was only available to the 24-hour unblinding system and the independent statistician providing analyses to the Data Safety Monitoring Board. Each participating country had a study monitor that was responsible for overview and monitoring of trial activities at each study site within that country.
