# Supplementary material for: Nilvadipine in mild to moderate Alzheimer disease: A randomised controlled trial
Source: PLoS Med. 2018 Sep 24;15(9):e1002660. doi: 10.1371/journal.pmed.1002660 (PMC6152871; doi:10.1371/journal.pmed.1002660)
Supplement: S2 Text — (DOCX) [file pmed.1002660.s002.docx]

## Detailed statistical methods

The primary analysis was specified as a model of change from baseline, with an unstructured residual correlation matrix and random intercepts for Country. Missing data was handled at the patient-visit level by rescaling scores if ≥80% of the possible maximum score was evaluable, or deleting otherwise. No other imputation was applied.

The mixed model for steps 1 (ADAS-Cog 12) and 2 (CDR-sb) included visit (3 levels corresponding to assessments at weeks 13, 52 and 78), treatment, treatment-by-visit interaction as the primary effect to establish efficacy, and the baseline endpoint scale as a covariate. The third gated step was identical, except for the use of continuous time rather than categorical visit, and omission of baseline endpoint as a covariate as this was included in the dependent variable. This allowed a precise time to be assigned to each assessment and also assumed that the group difference in change over time is linear.

Empty linear mixed models yielded intraclass correlation coefficients of 0.06 (bootstrap 95% confidence interval: 0.04, 0.07) for the ADAS-Cog 12, 0.07 (0.06, 0.08) for the CDR-sb, and 0.05 (0.04, 0.07) for the DAD.

Further exploratory analyses specified in the statistical analysis plan, and in a white paper developed by the steering committee prior to data lock, consisted of sensitivity to Country effects, dichotomous responder analysis, and the three pre-specified subgroup analyses using severity (SMMSE <20), sex, and APOE (ε4 allele carrier versus non-carrier).

The multiple significance tests that would have arisen from the separate outcome measures were dealt with using a gated approach. This method requires that, prior to the analysis, the outcomes are ordered in importance and the analysis is performed sequentially, starting with the first outcome. If this first analysis achieves a p-value less than 0.05, the next outcome can be analyzed. If that achieves p < 0.05, a significant effect of the first two outcomes is claimed and the third outcome is analyzed. This process continues until an outcome analysis gives a p > 0.05. This particular outcome is declared non-significant and all subsequent outcomes, irrespective of their p-values, must also be declared non-significant. This procedure, where each analysis acts as a gatekeeper for the next, preserves an overall Type I error of 5% without any adjustment of the actual p-values for multiple testing. The cost is that the first non-significant result stops further analysis and the remainder of the comparisons in the hierarchy are declared non-significant with only effect sizes estimated and no subsequent p-values computed.
