# Supplementary material for: Nilvadipine in mild to moderate Alzheimer disease: A randomised controlled trial
Source: PLoS Med. 2018 Sep 24;15(9):e1002660. doi: 10.1371/journal.pmed.1002660 (PMC6152871; doi:10.1371/journal.pmed.1002660)
Supplement: S3 Text — (DOCX) [file pmed.1002660.s003.docx]

## Trial-Associated Boards

Data Safety and Monitoring Committee

Peter Passmore, Professor of Geriatric Medicine, Queens University, Belfast Northern Ireland (Chair)

Bernadette McGuiness, Senior Lecturer Geriatric Medicine, Queens University, Belfast, Northern Ireland

John Newell, Professor in Biostatistics, National University of Ireland Galway

Martin O’Donnell Professor of Geriatric Medicine, National University of Ireland, Galway

Ethics Advisory Committee

Dr. Shaun O’ Keeffe, Consultant Geriatrician, University Hospital Galway (Chair)

Dr. Tony O’ Brien, Palliative Care Consultant, Marymount Hospice, Cork

Dr. Mary Donnelly, School of Law, University College, Cork

Ursula Collins, Alzheimer’s Society of Ireland

List of National Competent Authorities

Ireland = HPRA <http://www.hpra.ie>

UK = MHRA <http://www.mhra.gov.uk>

France = ANSM http://www.ansm.sante.fr/

Germany = BFARM [https://www.bfarm.de/DE/Home/home_node.html](https://mail.stjames.ie/owa/14.3.319.2/scripts/premium/redir.aspx?C=TLp8SPJPAlZNcwOocWHlkdOl-3lyba51R733aPtuweMszwNays7VCA..&URL=https%3a%2f%2fwww.bfarm.de%2fDE%2fHome%2fhome_node.html)

Italy = AIFA [http://www.agenziafarmaco.gov.it/en](https://mail.stjames.ie/owa/14.3.319.2/scripts/premium/redir.aspx?C=immxsUJOwsCWnqG65p6sUczudoz3_PQIQ6-KcCqBqI0szwNays7VCA..&URL=http%3a%2f%2fwww.agenziafarmaco.gov.it%2fen)

Sweden = Lakemedelsverket [https://lakemedelsverket.se/](https://mail.stjames.ie/owa/14.3.319.2/scripts/premium/redir.aspx?C=KFdLHQq59EFUS60SvoGm_7W9TvD9nyAavBO4iceWXbIszwNays7VCA..&URL=https%3a%2f%2flakemedelsverket.se%2f)

Hungary = OGYEI [https://www.ogyei.gov.hu/nyitooldal/](https://mail.stjames.ie/owa/14.3.319.2/scripts/premium/redir.aspx?C=FM2HsGTMul8PKy0Dsff6CWFb4ojBJ3bqKLLxCt11aWkszwNays7VCA..&URL=https%3a%2f%2fwww.ogyei.gov.hu%2fnyitooldal%2f)

Greece= National Organisation for Medicines [http://www.eof.gr/web/guest;jsessionid=015547c32e251f9f4bca4c3ee9fe](https://mail.stjames.ie/owa/14.3.319.2/scripts/premium/redir.aspx?C=eIb8tZRUcMQVv84-4ezSHcj0xKwGGVbl93V3_LNLJmIszwNays7VCA..&URL=http%3a%2f%2fwww.eof.gr%2fweb%2fguest%3bjsessionid%3d015547c32e251f9f4bca4c3ee9fe)

Netherlands**=** Medicines Evaluation Board [https://www.cbg-meb.nl/](https://mail.stjames.ie/owa/14.3.319.2/scripts/premium/redir.aspx?C=7IYeBx4A5PLtN4yY1bJkLj0cibeaUYCrkTSd1zLsAqgszwNays7VCA..&URL=https%3a%2f%2fwww.cbg-meb.nl%2f)

| **List of IECs** | | |
| --- | --- | --- |
| **Country** | **Site** | **IEC** |
| France | CHU Amiens | Comité de Protection des Personnes Nord Ouest III (Chair: Mme Charlotte Gourio) |
|  | CH Bethune |  |
|  | CHU Caen |  |
|  | CH Calais |  |
|  | CH Saint-Philibert, GHICL |  |
|  | CHRU Lille |  |
|  | CH Lens |  |
| Greece | Pagageorgiou General Hospital | Scientific Council of Papanikolaou Hospital Thessaloniki |
|  | Papanikolaou General Hospital of Thessaloniki |  |
|  | AXEPA University General Hospital |  |
| Holland | Rijnstate Hospital, Arnhem | Radboud universitair medisch centrum Concernstaf Kwaliteit en Veiligheid Commissie Mensgebonden Onderzoek Regio Arnhem-Nijmegen (Chair: M.J.J. Prick) |
|  | Academic Hospital, Maastricht |  |
|  | Radboud University Medical Centre, Nijmegen |  |
| Hungary | University of Szeged | Medical Research Council Ethics Committee for Clinical Pharmacology (KFEB) (Chair: Dr Fϋrst Zsuzsarina) |
| Italy | IRCCS Centro san Giovanni di Dio-Fatebenefratelli Brescia | C E I O C – Comitato Etico Istituzioni Ospedaliere Cattoliche (Chair: Dr. Giovanni Zaninetta) |
|  | IRCCS Multimedica Castellanza | Comitato Etico IRCCS MultiMedica (Chair: Prof Emilio Trabucchi) |
|  | IRCSS AOU San Martino Genoa | Comitato Etico dell’Aienda Ospedaliera Universitaria S. Martino di Genova (Chair: Dott. Luigi Francesco Meloni)  and  Comitato Etico Regione Liguria (Chair: Prof. Fulvio Brema) |
|  | Fondazione Don Gnocchi  Milan | Comitato Etico Fondazione Don Carlo Gnocchi (Chair: Prof Flaminio Cattabeni) |
