# Supplementary material for: Nilvadipine in mild to moderate Alzheimer disease: A randomised controlled trial
Source: PLoS Med. 2018 Sep 24;15(9):e1002660. doi: 10.1371/journal.pmed.1002660 (PMC6152871; doi:10.1371/journal.pmed.1002660)
Supplement: S4 Text — (DOCX) [file pmed.1002660.s004.docx]

## Personal correspondence

Excerpt from correspondence with J Claassen, following up on discussions with MGM Olde Rikkert concerning the balance of blood pressure medication other than nilvadipine between arms in the NILVAD trial:

**From:** [Marcel.OldeRikkert@radboudumc.nl](mailto:Marcel.OldeRikkert@radboudumc.nl) [[Marcel.OldeRikkert@radboudumc.nl](mailto:Marcel.OldeRikkert@radboudumc.nl)]
**Sent:** Tuesday, July 31, 2018 8:33 AM
**To:** Lawlor, Brian (Consultant Psychiatrist)
**Cc:** [Jurgen.Claassen@radboudumc.nl](mailto:Jurgen.Claassen@radboudumc.nl); [Rianne.deHeus@radboudumc.nl](mailto:Rianne.deHeus@radboudumc.nl)
**Subject:** RE: Personal communication

Dear Brian,

I am happy to respond on this on behalf of Jurgen, as he has just left for holidays, and will not reply till late in August.

The data of our substudy confirm that there is no relevant nor significant imbalance between the Nilvadipine group and the placebo.

This holds true  for drug use at the start of the trial and for changes during the trial.
