# Supplementary material for: Nilvadipine in mild to moderate Alzheimer disease: A randomised controlled trial
Source: PLoS Med. 2018 Sep 24;15(9):e1002660. doi: 10.1371/journal.pmed.1002660 (PMC6152871; doi:10.1371/journal.pmed.1002660)
Supplement: S1 Table — (DOCX) [file pmed.1002660.s005.docx]

**S1 Table. Distribution of treatment arms recruited in each site**

| Country | Site | Nilvadipine (N=247) | Placebo (N=251) |
| --- | --- | --- | --- |
| France | 11 | 2 | 2 |
|  | 12 | 4 | 3 |
|  | 13 | 2 | 3 |
|  | 14 | 4 | 4 |
|  | 15 | 0 | 1 |
|  | 16 | 11 | 12 |
|  | 17 | 4 | 4 |
| Greece | 31 | 4 | 3 |
|  | 32 | 40 | 41 |
|  | 33 | 4 | 5 |
| Netherlands | 41 | 6 | 6 |
|  | 42 | 7 | 8 |
|  | 43 | 24 | 26 |
| Hungary | 51 | 7 | 7 |
| Italy | 61 | 7 | 7 |
|  | 62 | 6 | 5 |
|  | 63 | 7 | 7 |
|  | 64 | 7 | 7 |
| Sweden | 71 | 10 | 10 |
| UK | 81 | 32 | 31 |
| Ireland | 82 | 19 | 20 |
|  | 83 | 36 | 35 |
| Germany | 91 | 4 | 4 |
