# Supplementary material for: Nilvadipine in mild to moderate Alzheimer disease: A randomised controlled trial
Source: PLoS Med. 2018 Sep 24;15(9):e1002660. doi: 10.1371/journal.pmed.1002660 (PMC6152871; doi:10.1371/journal.pmed.1002660)
Supplement: S2 Table — ADAS-Cog 12, Alzheimer's Disease Assessment Scale Cognitive-12. (DOCX) [file pmed.1002660.s006.docx]

**S2 Table. ADAS-Cog 12 Sex subgroup analysis results**

|  | | **Visit** | | | |
| --- | --- | --- | --- | --- | --- |
|  |  | Week 0 | Week 13 | Week 52 | Week 78 |
| Male (N=190) | Nilvadipine | 32.5 ± 10.5 | 33.8 ± 10.4 | 35.8 ± 11.7 | 38.4 ± 12.3 |
|  | Δ (95% CI) |  | 1.31 (-0.01, 2.62) | 4.75 (2.96, 6.53) | 8.84 (6.64, 11.04) |
|  | Placebo | 34.6 ± 11.5 | 35.4 ± 12.3 | 41.8 ± 14.2 | 42.6 ± 14.6 |
|  | Δ (95% CI) |  | 0.82 (-0.40, 2.05) | 7.63 (6.01, 9.26) | 10.80 (8.78, 12.81) |
|  | *Group difference* |  | *0.48 (-1.25, 2.21)* | *-2.89 (-5.26, -0.51)* | *-1.95 (-4.91, 1.00)* |
| Female (N=308) | Nilvadipine | 35.4 ± 10.4 | 36.0 ± 11.7 | 41.3 ± 13.4 | 43.8 ± 15.4 |
|  | Δ (95% CI) |  | 0.66 (-0.36, 1.67) | 6.29 (4.96, 7.62) | 9.71 (8.09, 11.32) |
|  | Placebo | 34.5 ± 10.3 | 35.2 ± 11.1 | 39.3 ± 13.7 | 41.4 ± 14.4 |
|  | Δ (95% CI) |  | 0.76 (-0.29, 1.81) | 5.58 (4.22, 6.94) | 8.86 (7.20, 10.52) |
|  | *Group difference* |  | *-0.10 (-1.45, 1.25)* | *0.71 (-1.13, 2.54)* | *0.84 (-1.42, 3.11)* |

Figures represent crude Mean ± Standard deviations and model-derived Δ (change from baseline) and group differences, with 95% confidence intervals, adjusted for Week 0 ADAS-Cog 12 and random intercepts for Country. Note: negative figures for the Group difference indicate less decline on nilvadipine
