# Supplementary material for: Nilvadipine in mild to moderate Alzheimer disease: A randomised controlled trial
Source: PLoS Med. 2018 Sep 24;15(9):e1002660. doi: 10.1371/journal.pmed.1002660 (PMC6152871; doi:10.1371/journal.pmed.1002660)
Supplement: S3 Table — ε4, epsilon 4 allele; ADAS-Cog 12, Alzheimer's Disease Assessment Scale Cognitive-12; APOE, Apolipoprotein E gene. (DOCX) [file pmed.1002660.s007.docx]

**S3 Table. ADAS-Cog 12 APOE ε4 Subgroup analysis results**

|  | | **Visit** | | | |
| --- | --- | --- | --- | --- | --- |
|  |  | Week 0 | Week 13 | Week 52 | Week 78 |
| Non-carrier (N=135) | Nilvadipine | 35.8 ± 11.1 | 37.1 ± 12.4 | 42.9 ± 14.1 | 44.6 ± 15.2 |
|  | Δ (95% CI) |  | 1.37 (-0.16, 2.90) | 8.11 (6.04, 10.18) | 11.40 (8.88, 13.93) |
|  | Placebo | 37.2 ± 11.6 | 38.1 ± 12.2 | 43.0 ± 12.8 | 46.7 ± 14.5 |
|  | Δ (95% CI) |  | 0.82 (-0.75, 2.40) | 5.79 (3.71, 7.87) | 10.47 (7.96, 12.98) |
|  | *Group difference* |  | *0.55 (-1.51, 2.60)* | *2.32 (-0.53, 5.17)* | *0.93 (-2.56, 4.43* |
| Carrier (N=194) | Nilvadipine | 33.3 ± 10.4 | 34.5 ± 10.3 | 37.5 ± 11.3 | 40.1 ± 12.8 |
|  | Δ (95% CI) |  | 1.14 (-0.19, 2.48) | 5.08 (3.27, 6.88) | 8.65 (6.48, 10.83) |
|  | Placebo | 33.9 ± 10.1 | 34.1 ± 10.7 | 40.7 ± 14.2 | 41.0 ± 14.0 |
|  | Δ (95% CI) |  | 0.20 (-1.13, 1.54) | 6.87 (5.17, 8.57) | 9.98 (7.93, 10.03) |
|  | *Group difference* |  | *0.94 (-0.76, 2.65)* | *-1.79 (-4.17, 0.59)* | *-1.33 (-4.24, 1.59)* |

Figures represent crude Mean ± Standard deviations and model-derived Δ (change from baseline) and group differences, with 95% confidence intervals, adjusted for Week 0 ADAS-Cog 12 and random intercepts for Country. Note: negative figures for the Group difference indicate less decline on nilvadipine
