# Supplementary material for: Nilvadipine in mild to moderate Alzheimer disease: A randomised controlled trial
Source: PLoS Med. 2018 Sep 24;15(9):e1002660. doi: 10.1371/journal.pmed.1002660 (PMC6152871; doi:10.1371/journal.pmed.1002660)
Supplement: S4 Table — ADAS-Cog 12, Alzheimer's Disease Assessment Scale Cognitive-12. (DOCX) [file pmed.1002660.s008.docx]

**S4 Table. ADAS-Cog 12 Severity subgroup analysis results**

|  | | **Visit** | | | |
| --- | --- | --- | --- | --- | --- |
|  |  | Week 0 | Week 13 | Week 52 | Week 78 |
| Mild (N=187) | Nilvadipine | 29.3 ± 7.1 | 29.8 ± 8.0 | 33.2 ± 9.4 | 35.3 ± 10.6 |
|  | Δ (95% CI) |  | -0.04 (-1.14, 1.05) | 3.26 (1.89, 4.62) | 5.76 (4.19, 7.34) |
|  | Placebo | 29.5 ±7.6 | 30.2 ± 8.8 | 34.5 ± 10.6 | 36.8 ± 12.4 |
|  | Δ (95% CI) |  | 0.05 (-1.05, 1.14) | 4.91 (3.55, 6.26) | 7.59 (6.03, 9.16) |
|  | *Group difference* |  | *-0.09 (-1.40, 1.23)* | *-1.69 (-3.45, 0.06)* | *-1.86 (-3.94, 0.23)* |
| Moderate (N=311) | Nilvadipine | 42.9 ± 9.7 | 44.3 ± 10.3 | 50.7 ± 11.2 | 55.9 ± 11.9 |
|  | Δ (95% CI) |  | 2.58 (1.19, 3.97) | 10.33 (8.54, 12.13) | 16.41 (14.25, 18.56) |
|  | Placebo | 42.9 ±10.0 | 44.0 ± 10.6 | 50.3 ± 13.2 | 52.5 ± 12.8 |
|  | Δ (95% CI) |  | 2.23 (0.83, 3.62) | 9.09 (7.35, 10.84) | 13.35 (11.24, 15.47) |
|  | *Group difference* |  | *0.36 (-1.34, 2.06)* | *1.24 (-1.08, 3.56)* | *3.05 (0.18, 5.93)* |

Severity defined as Mild for baseline SMMSE score ≥20, and Moderate for SMMSE <20.

Figures represent crude Mean ± Standard deviations and model-derived Δ (change from baseline) and group differences, with 95% confidence intervals, adjusted for Week 0 ADAS-Cog 12 and random intercepts for Country. Note: negative figures for the Group difference indicate less decline on nilvadipine
