# Supplementary material for: Nilvadipine in mild to moderate Alzheimer disease: A randomised controlled trial
Source: PLoS Med. 2018 Sep 24;15(9):e1002660. doi: 10.1371/journal.pmed.1002660 (PMC6152871; doi:10.1371/journal.pmed.1002660)
Supplement: S5 Table — (DOCX) [file pmed.1002660.s009.docx]

**S5 Table.** **Clinical Chemistry and Haematology findings at screening visit and week 78**

| **Marker with Abnormal results** | **Screening** | | **Week 78** | |
| --- | --- | --- | --- | --- |
|  | Nilvadipine | Placebo | Nilvadipine | Placebo |
| *Clinical Chemistry* | | | | |
| Sodium | 9 (4%) | 8 (3%) | 5 (3%) | 3 (1%) |
| Potassium | 10 (4%) | 10 (4%) | 7 (4%) | 8 (4%) |
| Chloride | 12 (5%) | 16 (6%) | 12 (6%) | 17 (9%) |
| Glucose | 65 (26%) | 37 (14%) | 60 (31%) | 44 (22%) |
| BUN | 33 (13%) | 39 (15%) | 25 (13%) | 34 (16%) |
| Creatinine | 42 (17%) | 24 (9%) | 23 (12%) | 27 (13%) |
| Calcium | 28 (11%) | 18 (7%) | 10 (5%) | 22 (11%) |
| Total Protein | 13 (5%) | 7 (3%) | 8 (4%) | 13 (6%) |
| Albumin | 14 (6%) | 14 (6%) | 21 (11%) | 18 (9%) |
| Bilirubin | 14 (6%) | 18 (7%) | 5 (3%) | 16 (8%) |
| Alkaline Phosphatase | 22 (9%) | 23 (9%) | 15 (8%) | 13 (6%) |
| Alanine Aminotransferase | 6 (2%) | 6 (2%) | 5 (3%) | 7 (3%) |
| Aspartate Aminotransferase | 10 (4%) | 9 (4%) | 3 (2%) | 6 (3%) |
| eGFR | 56 (22%) | 53 (21%) | 40 (21%) | 44 (21%) |
| *Haematology* | | | | |
| White Blood Cell count | 13 (5%) | 15 (6%) | 8 (4%) | 12 (6%) |
| Red Blood Cell count | 41 (16%) | 39 (15%) | 31 (16%) | 30 (14%) |
| Haemoglobin (Hb) | 36 (14%) | 26 (10%) | 22 (11%) | 32 (15%) |
| Packed cell volume | 37 (15%) | 43 (17%) | 29 (15%) | 31 (15%) |
| Microtic cell volume | 24 (10%) | 31 (12%) | 13 (7%) | 24 (12%) |
| Mean Corpuscular Hb (MCH) | 13 (5%) | 15 (6%) | 10 (5%) | 14 (7%) |
| MCH concentration | 19 (8%) | 17 (7%) | 18 (9%) | 20 (10%) |
| Platelets | 11 (4%) | 7 (3%) | 11 (6%) | 4 (2%) |

Laboratory findings were classified (according to local criteria) as normal, abnormal (not significant) or abnormal (significant), however the latter comprised at most 1-3 cases per assay, and were never ‘significant’ for 46/56 tests per group/time. The table below therefore shows the number and percentage of patient samples with collapsed ‘abnormal’ results. Note that denominators vary due to missed sampling or failed assays.
